# Inhibition of Protease-Activated Receptor 1 Does not Affect Dendritic Homeostasis of Cultured Mouse Dentate Granule Cells

**DOI:** 10.3389/fnana.2016.00064

**Published:** 2016-06-13

**Authors:** Gerlind Schuldt, Christos Galanis, Andreas Strehl, Meike Hick, Sabine Schiener, Maximilian Lenz, Thomas Deller, Nicola Maggio, Andreas Vlachos

**Affiliations:** ^1^Institute of Clinical Neuroanatomy, Neuroscience Center, Goethe-University FrankfurtFrankfurt, Germany; ^2^Institute of Anatomy II, Faculty of Medicine, Heinrich-Heine-University DüsseldorfDüsseldorf, Germany; ^3^Department of Neurology, The Sagol Center for Neurosciences, Sheba Medical Center, Affiliated to the Sackler Faculty of Medicine, Tel Aviv UniversityTel Aviv, Israel; ^4^Talpiot Medical Leadership Program, Department of Neurology and J. Sagol Neuroscience Center, The Chaim Sheba Medical CenterTel HaShomer, Israel; ^5^Sagol School of Neuroscience, Tel Aviv UniversityTel Aviv, Israel

**Keywords:** hippocampus, entorhinal cortex lesion, brain injury, coagulation, thrombin

## Abstract

Protease-activated receptors (PARs) are widely expressed in the central nervous system (CNS). While a firm link between PAR1-activation and functional synaptic and intrinsic neuronal properties exists, studies on the role of PAR1 in neural structural plasticity are scarce. The physiological function of PAR1 in the brain remains not well understood. We here sought to determine whether prolonged pharmacologic PAR1-inhibition affects dendritic morphologies of hippocampal neurons. To address this question we employed live-cell microscopy of mouse dentate granule cell dendrites in 3-week old entorhino-hippocampal slice cultures prepared from Thy1-GFP mice. A subset of cultures were treated with the PAR1-inhibitor SCH79797 (1 μM; up to 3 weeks). No major effects of PAR1-inhibition on static and dynamic parameters of dentate granule cell dendrites were detected under control conditions. Granule cells of PAR1-deficient slice cultures showed unaltered dendritic morphologies, dendritic spine densities and excitatory synaptic strength. Furthermore, we report that PAR1-inhibition does not prevent dendritic retraction following partial deafferentation *in vitro*. Consistent with this finding, no major changes in PAR1-mRNA levels were detected in the denervated dentate gyrus (DG). We conclude that neural PAR1 is not involved in regulating the steady-state dynamics or deafferentation-induced adaptive changes of cultured dentate granule cell dendrites. These results indicate that drugs targeting neural PAR1-signals may not affect the stability and structural integrity of neuronal networks in healthy brain regions.

## Introduction

Protease activated receptors (PAR) are G-protein coupled receptors, which are activated by proteolytic cleavage of their N-terminal extracellular domain (Gingrich and Traynelis, 2000; Traynelis and Trejo, 2007; Zhao et al., 2014). This modification uncovers a tethered ligand, which activates intracellular Ga_q/11_, Ga_i/o_, or Ga_12/13_ proteins (Coughlin, 2000; Macfarlane et al., 2001; Traynelis and Trejo, 2007). While PAR2 represents a class of trypsin/tryptase-activated receptors, PAR1, PAR3, and PAR4 are effectively activated by thrombin (Gingrich and Traynelis, 2000). Among other PAR-activating proteases are plasmin, activated factor X, activated protein C, and matrix metalloproteinases (Mosnier et al., 2012; Austin et al., 2013; Gleeson et al., 2014). In the vascular system PARs mediate important biological functions, such as blood coagulation, vascular integrity, and inflammation (Alberelli and De Candia, 2014; Hollenberg et al., 2014). In the brain, PAR1 is detected in the cerebral cortex, hippocampus, amygdala, basal ganglia, and striatum (Weinstein et al., 1995; Niclou et al., 1998; Striggow et al., 2001; Junge et al., 2004; Han et al., 2011). Inhibition of PAR1 has been comprehensively studied for its neuroprotective effects in various models of brain injury (Striggow et al., 2001; Junge et al., 2003; Guo et al., 2004; Olson et al., 2004; Nicole et al., 2005; Hamill et al., 2007; Isaev et al., 2015). Furthermore, recent studies revealed that the activation of PAR1 increases neural excitability and affects the ability of neurons to express synaptic plasticity (Ben Shimon et al., 2015). Likewise, evidence has been provided that PAR1-activation triggers neurite retraction (Jalink et al., 1994; Pai and Cunningham, 2002).

These findings are of considerable interest in the context of neurological diseases, which are accompanied by a break-down of the blood brain barrier (Rosenberg, 2012; Obermeier et al., 2013) and/or increased brain serine protease levels (Zhao et al., 2006, 2007; Amantea et al., 2007). Since novel oral anticoagulants have been developed that target PAR1 (Morrow et al., 2012; Scirica et al., 2012; Bhandari and Mehta, 2014) or affect PAR1-signals indirectly by blocking thrombin or factor Xa-activity (Verheugt and Granger, 2015), it has been proposed that these drugs may exert their positive effects seen in patients also by preventing the activation of neural PAR1 under pathological conditions (e.g., Luo et al., 2007; Maggio et al., 2013a). Hence, it has been speculated that neural PAR1-inhibitors may prove suitable for the treatment of several neurological diseases associated with alterations in blood-brain barrier function and/or increased PAR1-activity (Boven et al., 2003; Ishida et al., 2006; Rosenberg, 2012). However, the role of PAR1 in normal brain function remains not well understood.

We here employed 3-week old (≥18 days *in vitro*, DIV) entorhino-hippocampal slice cultures prepared from Thy1-GFP mice (Feng et al., 2000) to study the effects of prolonged pharmacologic PAR1-inhibition on static and dynamic properties of mature dentate granule cell dendrites. Live-cell microscopy was used to follow the dynamics of dendritic changes in identified GFP-expressing mature dentate granule cells over a period of up to 3 weeks *in vitro*. In addition, we tested whether PAR1 mediates dendritic retraction in response to entorhinal cortex lesion *in vitro* (Müller et al., 2010; Vlachos et al., 2012, 2013a), which is a strong stimulus for neurons to remodel their dendrites (Steward, 1994; Deller and Frotscher, 1997; Perederiy and Westbrook, 2013).

## Materials and Methods

### Ethics Statement

Mice were maintained in a 12 h light/dark cycle with food and water available *ad libitum*. Every effort was made to minimize distress of animals. All experimental procedures were performed according to German animal welfare legislation and approved by the animal welfare officer of Goethe-University Frankfurt, Faculty of Medicine.

### Preparation of Slice Cultures

Entorhino-hippocampal slice cultures were prepared at postnatal day 4–5 from heterozygous Thy1-GFP mice (Feng et al., 2000) and C57BL/6J mice of either sex as previously described (e.g., Vlachos et al., 2013b; Lenz et al., 2016). In some experiments PAR1-deficient mice (PAR1-KO; B6.129S4-F2r^tm1Ajc^/J; from Jackson Laboratories, USA) and their wild type littermates were used (c.f., Becker et al., 2013). Each filter inset contained up to six cultures prepared from one animal. Cultures from at least three independent litters were used in the experiments. Cultivation medium contained 50% (v/v) MEM, 25% (v/v) basal medium eagle, 25% (v/v) heat-inactivated normal horse serum, 25 mM HEPES buffer solution, 0.15% (w/v) bicarbonate, 0.65% (w/v) glucose, 0.1 mg/ml streptomycin, 100 U/ml penicillin, and 2 mM glutamax. The pH was adjusted to 7.3 and the medium was replaced three times per week. All slice cultures were allowed to mature for at least 18 days in humidified atmosphere with 5% CO_2_ at 35°C.

### Pharmacology

SCH79797 [IC_50_ = 70 nM; Tocris Bioscience, UK] was used to block PAR1. Tetrodotoxin (TTX) and D(-)-2-amino-5-phosphonovaleric acid (D-AP5; both from Tocris Bioscience, UK) were used for electrophysiological recordings. Handling and disposal of all drugs carried out in accordance to German and University regulations.

### Entorhinal Cortex Lesion

Slice cultures (18–20 DIV) were transected from the rhinal fissure to the hippocampal fissure using a sterile scalpel blade. The entorhinal cortex was removed from the culturing dish in every denervation experiment.

### Long-Term Time-Lapse Imaging of Slice Cultures

Live imaging of slice cultures was performed as described previously (e.g., Vlachos et al., 2012, 2013a). The imaging buffer contained 129 mM NaCl, 4 mM KCl, 1 mM MgCl_2_, 2 mM CaCl_2_, 4.2 mM glucose, 10 mM HEPES, 0.1 mM Trolox, 0.1 mg/ml streptomycin, 100 U/ml penicillin (pH 7.4; 35°C; osmolarity was adjusted with sucrose to match the osmolarity of the cultivation medium). Cultures (starting at 18–20 DIV) were viewed with an upright Zeiss LSM Pascal confocal microscope. A 10× water immersion objective (0.3 NA, Zeiss, Germany) was used to visualize the culture at low magnification to identify individual granule cells. Then a 40× water immersion objective (0.9 NA; Zeiss, Germany) was used to image the dendritic tree of a single granule cell. Up to 40 images were recorded per stack (512 × 512 pixel, 0.45 μm/pixel; *z*-steps: 3 μm). Per filter insert (containing up to six cultures, prepared from one animal) one identified granule cell was visualized to minimize dwell time during imaging procedure (<10 min per culture). The dendritic trees of individual GFP-expressing granule cells were repeatedly imaged for up to 3 weeks using the same imaging procedure and settings at the microscope.

### Reconstruction of the Dendritic Tree

The dendritic tree of individual imaged dentate granule cells was manually reconstructed in confocal image stacks using SpineLab 0.4 (Jungblut et al., 2012). In some cases single dendritic segments were excluded from the analysis because they were not completely mapped on single or several points of time. As reported previously the validity of reconstructions was confirmed by reconstructing a subset of neurons with Neurolucida Software 10.0 [MBF Bioscience; c.f., (Jungblut et al., 2012)]. Sholl analysis was performed on Neurolucida-reconstructed PAR1-deficient granule cells using the NeuroExplorer software (Neurolucida Software 10.0; MBF Bioscience) as described previously (Vuksic et al., 2011; Hick et al., 2015). In short, a series of concentric spheres were centered on the soma and mapped onto the 3D-reconstruction with increasing radius (40 μm increments). The number of reconstructed dendrites crossing each sphere was determined.

### Laser Capture Microdissection

Slice cultures were washed with phosphate buffered saline (PBS; 0.1 M, pH 7.4), shock frozen at −80°C in tissue freezing medium (Leica Microsystems, Germany), re-sliced into 10 μm thick slices on a cryostat (Leica CM 3050 S) and mounted on PET foil metal frames (Leica, Germany) as described previously (Becker et al., 2013, 2015). Re-sliced cultures were fixed in ice-cold acetone for 1 min and incubated with 0.1% (v/v) toluidine blue (Merck, Germany) at room temperature for 1 min to visualize cytoarchitecture, before rinsing in ultrapure water (DNase/RNase free, Invitrogen, USA) and 70% (v/v) ethanol. PET foil metal frames were mounted on a Leica DM 6000B laser capture microdissection (LMD) system (Leica Microsystems, Germany) with the section facing downward (Burbach et al., 2003). After adjusting intensity, aperture, and cutting velocity, the pulsed ultraviolet laser beam was carefully directed along the borders of the respective layers of the dentate gyrus (DG) using a 20× objective lens (Leica Laser Microdissection, Software Version 7.4.1.4853). Tissue was collected from the outer molecular layer (OML), the inner molecular layer (IML) and the granule cell layer (GCL) of the DG. Microdissected tissue was transferred by gravity into microcentrifuge tube caps placed underneath the sections, filled with 50 μl guanidine isothiocyanate (GITC)-containing buffer (RLT Buffer, RNeasy Mini Kit, Qiagen, Germany) with 1% (v/v) β-mercaptoethanol (AppliChem GmbH, Germany). Successful tissue collection was verified by visually inspecting the content of the tube caps. All samples were frozen and stored at −80°C.

### Isolating RNA and qPCR

RNA was isolated using the RNeasy^®^ MicroPlus Kit (Qiagen, Germany). Purified RNA was transcribed into cDNA with the High Capacity cDNA Reverse Transcription Kit (Applied Biosystems, MA, USA). All kits and assays were used according to the manufacturer’s instructions. The cDNA was amplified using the TaqMan^®^ PreAmp Master Mix Kit (Applied Biosystems, MA, USA) using 5 μl PreAmp Master Mix (Applied Biosystems, MA, USA) + 2.5 μl cDNA + 2.5 μl Assay Mix [TaqMan Gene Expression^TM^-Assay; GAPDH: 4352932E; PAR1: MM00438851_m1 from Applied Biosystems, MA, USA] with a standard amplification protocol (14 cycles: 95°C for 15 s, 60°C for 4 min). Amplified cDNAs were diluted 1:20 in ultrapure water and subjected to qPCR (StepOnePlus, Applied Biosystems, USA) using a standard amplification program (1 cycle of 50°C for 2 min, 1 cycle of 95°C for 10 min, 40 cycles of 95°C for 15 s and 60°C for 60 s cut off at 30 cycles; average C_T_-value PAR1: 26.5 ± 0.2 cycles).

### Whole-Cell Patch-Clamp Recordings

Whole-cell voltage-clamp recordings of miniature excitatory postsynaptic currents (mEPSCs; at 35°C) and *post hoc* identification of recorded neurons were carried out in slice cultures prepared from PAR1-deficient mice as previously described (e.g., Vlachos et al., 2013a). Briefly, mEPSCs were recorded in artificial cerebrospinal fluid (ACSF: 126 mM NaCl, 2.5 mM KCl, 26 mM NaHCO_3_, 1.25 mM NaH_2_PO_4_, 2 mM CaCl_2_, 2 mM MgCl_2_, and 10 mM glucose) in 10 μM D-AP5 and 0.5 μM TTX saturated with 95% O_2_/5% CO_2_. Patch pipettes contained 126 mM K-gluconate, 4 mM KCl, 4 mM ATP-Mg, 0.3 mM GTP-Na_2_, 10 mM PO-Creatine, 10 mM HEPES and 0.3% Biocytin (pH = 7.25 with KOH, 290 mOsm with sucrose). Dentate granule cells were recorded at a holding potential of −70 mV. Series resistance was monitored in 2 min intervals, and recordings were discarded if the series resistance and leak current changed significantly and/or reached ≥30 MΩ or ≥50 pA, respectively.

### Slice Culture Fixation, Staining and Imaging

Slice cultures were fixed in a solution of 4% (w/v) paraformaldehyde (PFA) in phosphate buffered saline (PBS, 0.1 M, pH 7.4) and 4% (w/v) sucrose for 1 h, followed by 2% PFA and 30% sucrose in PBS overnight. Fixed slice cultures were thoroughly washed and stained with Alexa568 coupled streptavidin (1:500) in PBS with 10% (v/v) normal horse serum and 0.1% (v/v) Triton X-100 for 2 h. Sections were washed again, transferred onto glass slides and mounted for visualization with anti-fading mounting medium. Confocal image stacks of dentate granule cells were acquired using a Nikon Eclipse C1si laser-scanning microscope with a 40× objective lens (NA 1.3, Nikon Instruments, Germany). In addition individual dendritic segments in the OML were imaged at 4× scan zoom using a 60× objective lens (NA 1.4, oil immersion, Nikon Instruments, Germany). Detector gain and amplifier were initially set to obtain pixel intensities within a linear range.

### Quantification and Statistics

Dendritic morphologies of cultured dentate granule cells treated with SCH79797 [1 μM] were compared to age- and time-matched granule cells of untreated, i.e., vehicle-treated cultures, which were imaged the same number of times using the same imaging protocol. In addition, age- and time-matched denervated dentate granule cells that were vehicle- or SCH79797-treated were assessed. 3D reconstructions were performed with the person analyzing dendritic morphologies blind to experimental condition. The dynamics of dendrites were assessed by determining changes in dendritic length of individual branches at consecutive points in time. The sum of elongation events and retraction events between two imaging sessions was normalized to the total reconstructed dendritic length at the respective point in time. The dendritic remodeling index was defined as mean elongation + mean retraction amount, divided by mean total dendritic length (TDL).

Dendritic spine numbers of Alexa568-streptavidin stained granule cells in PAR1-deficient preparations and their age- and time-matched wild type littermates were assessed using NeuroExplorer software (Neurolucida Software 10.0; MBF Bioscience; c.f., Hick et al., 2015). At least 60 μm of dendritic length from three different dendritic segments per neuron were analyzed in the OML of the dentate gyrus by an investigator blind to genotype.

qPCR-data were analyzed as described by Pfaffl (2001). GAPDH served as reference gene in this analysis. The qPCR assay efficiency was calculated with the StepOnePlus software (Applied Biosystems, MA, USA) based on a dilution series of five samples (whole hippocampi) for each assay. Since PAR1-mRNA levels were not significantly different in non-denervated controls at 18–20 DIV and 32 DIV these data were pooled and used for statistical comparisons (Kruskal-Wallis-test followed by Dunn’s *post hoc*-test, which takes multiple comparisons into account).

Electrophysiological data were analyzed using pClamp 10.2 (Axon Instruments, CA, USA) and MiniAnalysis (Synaptosoft, GA, USA) software. All events were visually inspected and detected by an investigator blind to experimental condition; 200–300 events were analyzed per recorded neuron.

Sample sizes were chosen according to initial pilot experiments and prior studies that used similar experimental approaches to detect changes in dendritic morphologies/dynamics, spine densities, mEPSC properties and mRNA levels (e.g., Vlachos et al., 2009; Becker et al., 2013; Willems et al., 2016). Data were analyzed using GraphPad Prism 6 (GraphPad Software). Statistical comparisons were made using non-parametric Mann-Whitney-test or the Kruskal-Wallis-test followed by Dunn’s *post hoc*-test.

*P*-values of less than 0.05 were considered significant. All values represent means ± standard error of the mean (sem). In the figures not significant differences are indicated with “NS”.

### Digital Illustrations

Confocal image stacks were exported as 2D-projections from the Zeiss LSM image browser and stored as TIF files. Figures were prepared using Photoshop graphics software (Adobe, CA, USA). Contrast and brightness was adjusted based on the reconstruction skeletons to improve visualization of reconstructed neurons in the 2D-projections.

## Results

### Pharmacologic Inhibition of PAR1 does not Affect Basic Morphological Parameters of Dentate Granule Cell Dendrites

**Figure 1:**
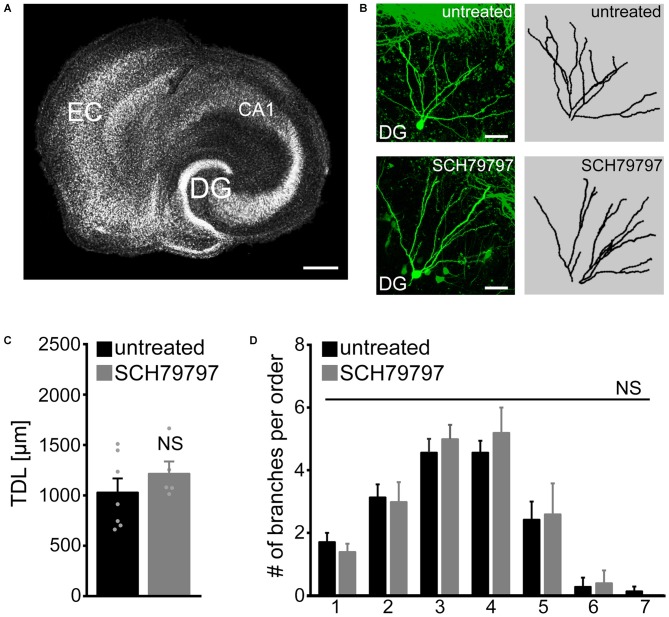
**Dendritic morphologies of cultured mouse dentate granule cells are not affected by pharmacologic protease-activated receptor (PAR)-inhibition under control conditions. (A)** Organotypic entorhino-hippocampal slice culture stained for TO-PRO^®^ nuclear stain. EC, entorhinal cortex; DG, dentate gyrus. Scale bar = 300 μm. **(B)** Individual GFP-expressing dentate granule cells are imaged in the suprapyramidal blade of the DG of 6-week-old entorhino-hippocampal slice cultures. Dendritic morphologies are reconstructed in 3D-image stacks. Scale bar = 30 μm. **(C)** Pharmacological inhibition of PAR1 for 3 weeks *in vitro* with SCH79797 [1 μM] does not affect total dendritic branch length (TDL) of cultured dentate granule cells. Untreated, *n* = 7 neurons; SCH79797, *n* = 5 neurons. Mann-Whitney-test (NS, not significant). **(D)** The mean number of dendritic segments per branch-order is not significantly different between the two groups. Kruskal-Wallis-test following Dunn’s *post hoc*-test (NS, not significant).

Dendritic trees of identified GFP-expressing granule cells were imaged in 6-week old entorhino-hippocampal slice cultures and reconstructed in 3D-image stacks (Figures 1A,B). TDL was determined in untreated control cultures and cultures that were treated for 3 weeks with SCH79797 (1 μM; treatment started 18–21 days after preparation), a potent and highly specific inhibitor of PAR1 (Ahn et al., 2000). No significant difference was detected between untreated and SCH79797-treated granule cells in these experiments (Figure 1C). We also compared the mean number of dendrites per dendritic branch order and did not find a significant difference between the two groups (Figure 1D). These initial results indicated that prolonged, i.e., 3-weeks of PAR1-inhibition does not cause major changes in TDL or dendritic branching of mature cultured mouse dentate granule cells.

### Time-Lapse Imaging Reveals the Dynamics of Granule Cell Dendrites in Untreated and SCH79797-Treated Slice Cultures

**Figure 2:**
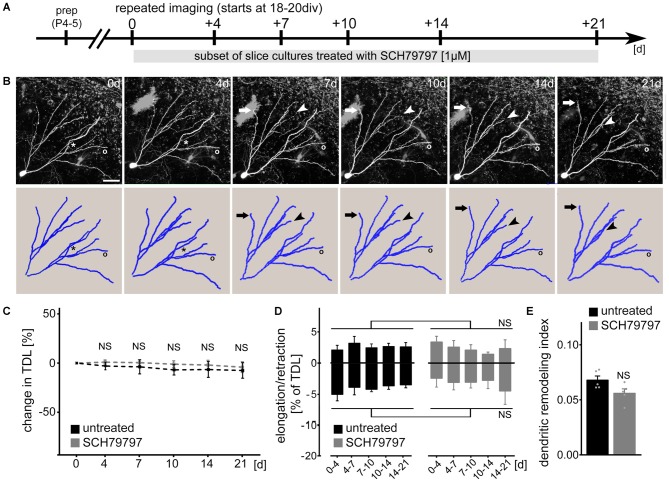
**PAR1-inhibition does not affect dendritic dynamics of cultured mouse dentate granule cells under control conditions. (A)** Schematic of the experimental design. Slice cultures prepared at postnatal day 4–5 are allowed to mature for 18–20 days *in vitro* (DIV). Repeated live-cell microscopy is performed at indicated points in time. A subset of cultures are treated with SCH79797 [1 μM] immediately after the first imaging session for the entire observation period. **(B)** SCH79797-treated dentate granule cell. Dynamic changes are predominantly (but not exclusively; see asterisk) observed at distal tips of dendrites. Arrow points at example of dendritic elongation, arrowhead at dendritic retraction, circle indicates a stable dendritic segment. Scale bar = 30 μm. **(C)** TDL of untreated and SCH79797-treated dentate granule cells at indicated points in time. Values normalized to TDL of the first imaging session, respectively. *n* = 5 neurons each. Kruskal-Wallis-test following Dunn’s *post hoc*-test (NS, not significant). **(D)** Elongation and retraction rates are comparable in the two groups. Kruskal-Wallis-test following Dunn’s *post hoc*-test (NS, not significant). **(E)** Accordingly, the dendritic remodeling index, i.e., sum of mean elongation and retraction amounts, divided by mean TDL, is not significantly different between the two groups. Approximately 5% of TDL (corresponding to an average of 64 ± 2 μm) is dynamically changed between consecutive imaging sessions. Mann-Whitney-test (NS, not significant).

Since the morphological parameters described above do not exclude the possibility that dynamic properties of dendrites are affected by pharmacologic PAR1-inhibition, we next employed live-cell microscopy to assess dendritic changes over time (Figure 2). Identified dentate granule cells were imaged repeatedly at selected points in time over a period of 3 weeks (between 3 and 6 weeks *in vitro*; Figure 2A) and their dendritic trees were reconstructed (Figure 2B). A set of cultures was treated with 1 μM SCH79797 (treatment started immediately after the first imaging session at 18–21 DIV). As shown in Figure 2C no significant difference in TDL was observed between the two groups. A slight trend towards reduced TDL was detected in these experiments, which, however, did not reach the level of significance in both groups (Kruskal-Wallis-test following Dunn’s *post hoc*-test; *p* = 0.17). We conclude from these experiments that TDL of dentate granule cells remains constant (between 3 and 6 weeks *in vitro*) in untreated control cultures and that pharmacologic inhibition of PAR1 has no major impact on TDL in our experimental setting (c.f., Figure 1).

We then quantified dendritic elongation and retraction between consecutive points in time in untreated and SCH79797-treated control cultures (Figure 2D). Dendritic changes were predominantly (but not exclusively) observed in distal portions of the dendritic tree (c.f., asterisk in Figure 2B). This analysis disclosed that mature, i.e., ≥18 DIV old granule cell dendrites are in a dynamic steady-state, in which elongation and retraction are in a homeostatic equilibrium. PAR1-inhibition did not affect the steady-state dynamics of cultured mouse granule cell dendrites. While TDL remained constant, the dendritic turnover index (sum of mean elongation and mean retraction amount, divided by mean TDL), was not significantly different between the two groups (Figure 2E). In both SCH79797-treated and untreated slice cultures approximately 5% of TDL (i.e., 64 ± 2 μm) was remodeled between consecutive points in time. We did not observe any dendritic segments to be completely lost or new segments to be formed during the observation period in both groups.

### SCH79797 does not Prevent Denervation-Induced Dendritic Retraction

**Figure 3:**
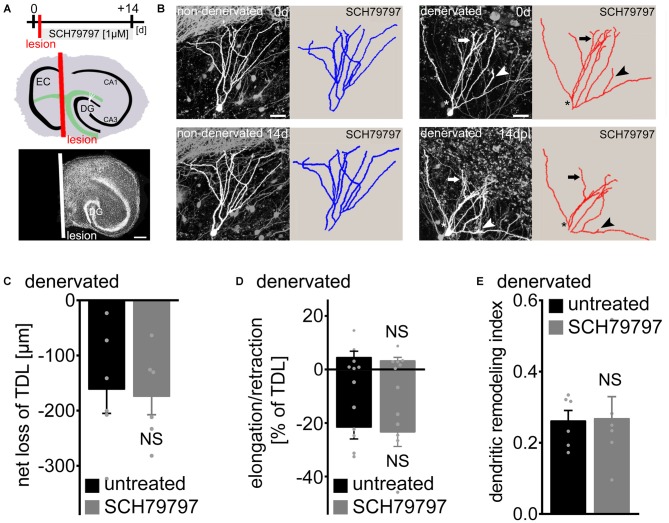
**Denervation-induced net retraction of cultured mouse dentate granule cell dendrites is not affected by PAR1-inhibition. (A)** Experimental design and schematic illustration of an organotypic entorhino-hippocampal slice culture (top). The entorhino-hippocampal fiber tract (green) terminating on distal portions of granule cell dendrites in the DG and the plane of transection (red) are illustrated (EC, entorhinal cortex). TO-PRO^®^ nuclear stain of a denervated slice culture. In all denervation experiments the EC is removed after the first imaging session and prior to pharmacological treatment using a sterile scalpel. Identified GFP-expressing dentate granule cells are imaged again 14 days later. Scale bar = 300 μm. **(B)** 2D-projected image stacks and corresponding reconstructions of Thy1-GFP expressing dentate granule cells imaged at 0 and 14 days post lesion (dpl). For comparison a non-denervated granule cell treated with the PAR1-inhibitor SCH79797 [1 μM] is illustrated at 0 and 14 days (c.f., Figure 2). Arrow points at example of dendritic elongation, arrowhead at dendritic retraction. Dendritic retraction is predominantly (but not exclusively; see asterisk) observed in distal portions of the dendritic tree. Scale bar = 30 μm. **(C)** Mean net loss of TDL in response to EC lesion *in vitro* is given for untreated and SCH79797-treated denervated dentate granule cells. *n* = 6 neurons in each group. Mann-Whitney-test (NS, not significant). **(D)** Elongation and retraction rates are not significantly different between the two groups. Mann-Whitney-test (NS, not significant). **(E)** The dendritic remodeling index, i.e., sum of elongation and retraction amounts divided by TDL, is not significantly different between the two groups. Approximately 25% of TDL (corresponding to 219 ± 30 μm) is remodeled between 0 and 14 dpl. This calculation underestimates the dynamics of granule cell dendrites, since elongation and retraction events occurring between the two imaging time-points escape detection. Mann-Whitney-test (NS, not significant).

To further assess the role of PAR1 in dendritic plasticity, we employed entorhinal denervation *in vitro* (Figure 3). Previous work has shown that the maintenance of granule cell dendrites requires entorhinal input (Caceres and Steward, 1983; Diekmann et al., 1996; Müller et al., 2010; Nitsch and Frotscher, 1993). Hence, in a different set of 3-week-old slice cultures the entorhino-hippocampal projection was transected to test for the effects of PAR1-inhibition on denervation-induced dendritic retraction (Figure 3A). The entorhinal cortex was removed from the culturing dish after the first imaging session and a subset of cultures were treated with SCH79797 [1 μM] immediately after the lesion for 14 days until the second imaging session (Figure 3A). The lesion does not damage the DG directly (Müller et al., 2010), but leads to the partial loss of innervating axons on distal portions of dentate granule cell dendrites. Identified GFP-expressing granule cells were imaged again 14 days later (Figures 3A,B). Indeed, a reduction in TDL was observed 14 days post lesion in untreated denervated cultures (Figure 3C). However, a similar effect of denervation on TDL was seen in the SCH79797-treated group (Figure 3C). Thus, PAR1-inhibition does not prevent the denervation-induced net reduction in TDL.

We then determined elongation and retraction rates and also calculated the dendritic remodeling index in denervated slice cultures (Figures 3D,E). No significant difference was detected between the two groups in this analysis. Denervation caused an increase in dendritic dynamics, with ~25% of TDL being remodeled between 0 and 14 days post lesion (Figure 3E). PAR1-inhibition had no apparent effect on the dynamic reorganization of dendrites: elongation and retraction of dendrites was similar in untreated and SCH79797-treated denervated cultures. We conclude that pharmacologic PAR1-inhibition does not affect denervation-induced dendritic adaptations of cultured dentate granule cells.

### No Major Changes in PAR1-mRNA Levels in the Denervated Dentate Gyrus

**Figure 4:**
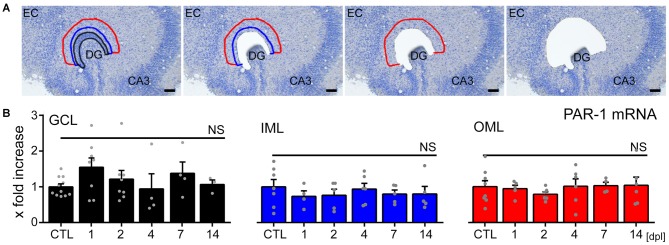
**No major changes in PAR1-mRNA levels are detected in the denervated DG of mouse entorhino-hippocampal slice cultures. (A)** Tissue from the granule cell layer (GCL; black), the inner molecular layer (IML; blue) and the denervated outer molecular layer (OML; red) was isolated by laser microdissection from denervated cultures at 1, 2, 4, 7, and 14 dpl and from non-denervated control cultures (CTL). Scale bar = 50 μm. **(B)** Isolated tissue was subjected to qPCR analysis. Values are normalized to non-denervated controls. No significant changes in PAR1-mRNA levels were observed in the denervated DG in response to EC lesion *in vitro*. *n* = 3–11 cultures per group. Kruskal-Wallis-test followed by Dunn’s *post hoc*-test (NS, not significant).

To test whether the loss of input triggers changes in PAR1-expression in the denervated zone, LMD combined with qPCR analysis was employed (Figure 4). Tissue isolated from the GCL, the IML and the denervated OML at 1, 2, 4, 7 and 14 dpl was subjected to qPCR analysis (Figure 4A). While PAR1-mRNA was robustly detected in all layers of the DG in our preparations, no significant changes in PAR1-mRNA levels were observed following entorhinal denervation *in vitro* (Figure 4B; a slight, but not significant increase was seen in the GCL during the early phase after denervation; Kruskal-Wallis-test following Dunn’s *post hoc*-test; *p* = 0.46). The results of these experiments disclosed that the loss of axonal input does not trigger major changes in PAR1-expression in the denervated DG of hippocampal slice cultures (c.f., Becker et al., 2013, 2015; on increased mRNA-levels of tumor necrosis factor and its receptors after denervation; Willems et al., 2016 on increased sphingosine-phosphate-receptor mRNA).

### Dentate Granule Cells of PAR1-Deficient Slice Cultures Show No Major Alterations in Dendritic Morphologies, Dendritic Spine Densities and Excitatory Synaptic Strength

**Figure 5:**
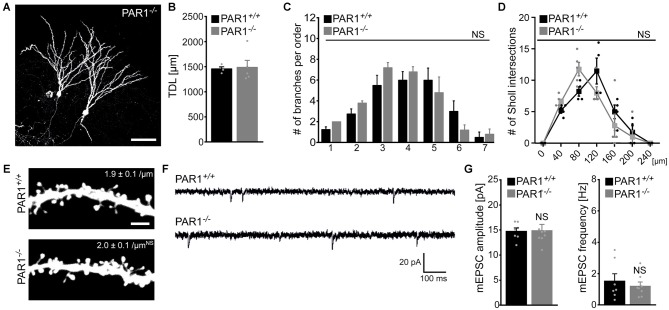
**Dendritic morphologies, spine density and excitatory synaptic currents of dentate granule cells prepared from PAR1-deficient mice. (A)** Examples of Alexa568-stained biocytin filled granule cells in slice cultures prepared from PAR1-deficient mice and wild type littermates. Dendritic morphologies were reconstructed in 3D-image stacks. Scale bar = 30 μm. **(B–D)** TDL **(B)**, the mean numbers of dendritic segments per branch-order **(B)** and Scholl-analysis **(C)** are not significantly different between the two groups. (*n* = 4 wild type neurons; *n* = 5 PAR1-deficient neurons; from three cultures each; Mann-Whitney-test in **B**; Kruskal-Wallis-test following Dunn’s *post hoc*-test in **C**; NS, not significant). **(E–G)** Dendritic spine densities in the outer molecular layer (OML) are comparable between the two groups (*n* = 6 neurons each group). Consistent with this finding no significant difference in miniature excitatory postsynaptic currents (mEPSCs) recorded from PAR1-deficient and wild type dentate granule is detected. (*n* = 7 wild type neurons; *n* = 8 PAR1-deficient neurons; from four cultures each; Mann-Whitney-test; NS, not significant).

Finally, slice cultures prepared from PAR1-deficient mice and their age- and time-matched wild type littermates were used to compare dendritic morphologies of dentate granule cells. Individual dentate granule cells were patched and filled with biocytin followed by Alexa568-Streptavidin staining (Figure 5A). As shown in Figures 5B–D, we did not observe any significant difference in TDL, the number of branches per order and number of Sholl intersections between the two groups. Interestingly, a trend towards higher TDL was observed in granule cells of both PAR1-deficient and wild type littermate cultures as compared to Thy1-GFP mice (c.f., Figure 1), which have a different genetic background. Although this difference did not reach the level of significance (Kruskal-Wallis-test following Dunn’s *post hoc*-test; *p* = 0.1), the observation underscores the importance of using wild type littermates when assessing dendritic morphologies of transgenic animals. We also assessed dendritic spine densities in the OML of PAR1-deficient and wild type cultures and did not find a significant difference between the two genotypes (Figure 5E). Consistent with these findings mEPSCs were unaltered in granule cells of PAR1-deficient preparations as compared to their age- and time-matched wild type littermates (Figures 5F,G; c.f., Becker et al., 2014). Altogether, these results supported our pharmacological experiments using 1 μM SCH79797, and, furthermore, suggested that PAR1-deficiency does not affect dendritic maturation of cultured dentate granule cells.

## Discussion

The present study addresses the effects of prolonged pharmacologic PAR1-inhibition in dendritic plasticity under control, i.e., steady-state conditions and following *in vitro* deafferentation. Our experiments disclose dynamics of dentate granule cell dendrites. Using live-cell microscopy of entorhino-hippocampal slice cultures prepared from Thy1-GFP mice we were able to demonstrate that granule cell dendrites are in a homeostatic, i.e., “stable dynamic state”: while TDL and dendritic branching remains constant, spontaneous dendritic remodeling is detected. This *dendritic homeostasis* is challenged by entorhinal denervation *in vitro*. As a consequence dendritic remodeling increases substantially, leading to a net reduction in TDL (see also Willems et al., 2016). Denervated neurons may eventually reach a new stable dynamic state at a later time point after the lesion (Caceres and Steward, 1983; Vuksic et al., 2011). PAR1-inhibition has no major effect on dendritic dynamics, both under control conditions and following partial denervation *in vitro*. Hence, our experiments suggest that PAR1 is not involved in maintaining TDL or dendritic branching under control conditions, nor is it linked to spontaneous or lesion-induced dendritic elongation and retraction events; a suggestion that is also supported by our LMD-qPCR results on unaltered PAR1-mRNA levels in the denervated DG. Consistent with these findings we did not observe any significant difference in dendritic morphologies (and spine densities) between dentate granule cells of PAR1-deficient and wild type littermate cultures. Although more subtle structural and functional alterations of dentate granule cells may have escaped our detection (e.g., Krueppel et al., 2011; Kamijo et al., 2014), these results suggest that PAR1 is not a major component of the homeostatic machinery that maintains dendrites under control conditions. PAR1 is not required to mediate the adjustment of dendritic morphologies in denervated brain regions.

### The Physiological Function of Neural PAR1 Remains Not Well Understood

Despite numerous studies, which have addressed the effects of PAR1-activation on neural excitability, synaptic plasticity and in the context of neuroprotection, the role of PAR1 for physiological brain functions remains not well understood. The majority of studies conducted in this field of research are based on experiments that employ pharmacologic activation of PAR1 or PAR1-inhibition under pathological conditions. It has been shown for example that PAR1-activation (with high concentration of thrombin or SFLLRN, a specific activating peptide) persistently increases neuronal sodium currents (Isaeva et al., 2012), modulates inhibitory synaptic transmission (Hashimotodani et al., 2011; Maggio et al., 2013b) and enhances NMDA-R activity (Gingrich et al., 2000; Lee et al., 2007; Maggio et al., 2008; Han et al., 2011; Becker et al., 2014; Vance et al., 2015). Furthermore, evidence has been provided that the activation of PAR1 occludes the ability of neurons to express synaptic plasticity under certain pathological conditions (Becker et al., 2014; Stein et al., 2015). While these results are relevant in diseases associated with increased neural PAR1-activity, they do not address the relevance of PAR1-signals in physiological brain function. In fact, the endogenous activators of PAR1 in the central nervous system (CNS) remain unknown (Kuliopulos et al., 1999; Nagai et al., 2006).

The physiological role of PAR1 in synaptic plasticity and memory formation has been recently addressed in a study using PAR1-deficient mice (Almonte et al., 2013). Indeed, alteration in Hebbian plasticity at Schaffer collateral-CA1 synapses and behavioral deficits in contextual fear conditioning were reported. These results are in favor of a role of neural PAR1 in learning and memory formation under physiological conditions. As discussed by Almonte et al. (2013), however, known limitations of models of constitutive gene deficiency cannot be ruled out. For example, developmental disturbances or compensatory mechanisms may have influenced the ability of neurons to express plasticity, rather than the lack of PAR1 *per se*. Notably, studies employing pharmacologic inhibition of PAR1 do not show major alterations in neuronal synaptic plasticity (Becker et al., 2014; Stein et al., 2015). Furthermore, PAR1-inhibition with SCH79797 [1 μM] has been shown to rescue the ability of neurons to express LTP at Schaffer-collateral CA1 synapses under conditions of increased PAR1-activity (Stein et al., 2015). The experiments of the present study reveal that 1 μM SCH79797 has no major effects on dendritic morphologies of cultured dentate granule cells. Furthermore, no alterations in dendritic morphologies, dendritic spine counts and excitatory synaptic strength are observed in granule cells of PAR1-deficient slice cultures. Apparently, more work is required to unravel the role of neural PAR1-activation under physiological conditions, and the precise molecular mechanisms of PAR1-mediated neural plasticity.

### PAR1 is Not Involved in Mediating Denervation-Induced Plasticity

Since no apparent effect of PAR1-inhibition on dendritic dynamics under control conditions was detected, we wondered whether PAR1 is involved in mediating dendritic retraction following deafferentation. This appeared to be a possibility, since earlier work demonstrated that thrombin-mediated PAR1-activation induces neurite retraction in neuroblastoma derived cell lines (Jalink et al., 1994; Pai and Cunningham, 2002). Accordingly, we employed *in vitro* entorhinal cortex lesion and expected that SCH79797 will prevent the denervation-induced dendritic retraction: we found a normal retraction response in these experiments. Both the net reduction in TDL and the increase in dendritic dynamics were not affected by the PAR1-inhibitor. In line with this observation, we did not observe major changes in PAR1-mRNA levels after denervation. These findings argue against a role of neural PAR1-activity in dendritic plasticity under control conditions and following deafferentation.

The results are in line with our previous work on the role of PAR1-inhibition in denervation-induced homeostatic synaptic plasticity (Becker et al., 2014). In this earlier study we were able to show that pharmacologic inhibition of PAR1 does not prevent the compensatory, i.e., homeostatic strengthening of granule cell excitatory synapses in response to entorhinal cortex lesion *in vitro* (Becker et al., 2014). Similarly, baseline synaptic transmission and denervation-induced homeostatic synaptic strengthening is not affected in granule cells of PAR1-deficient preparations (Becker et al., 2014). The results of the present study confirm and extend previous findings, since no significant difference in mEPSC amplitudes and frequencies was observed and spine densities were comparable in PAR1-deficient slice cultures and wild type littermates. Taken together, we propose that PAR1-inhibition has no major impact on the ability of neurons to adjust structural and functional properties of neurons in response to perturbations of network activity.

### Clinical Implications

Neurological diseases are often accompanied by neuronal cell death and widespread secondary changes in connected brain regions, which are mainly caused by the loss of innervating axons originating from neurons at the primary lesion site. This situation severely disrupts otherwise unaffected and healthy brain areas and perturbs network function (Steward, 1994; Deller and Frotscher, 1997; Perederiy and Westbrook, 2013; Sharp et al., 2014). Our results suggest that pharmacologic PAR1-inhibtion will not affect neural homeostasis, i.e., structural and functional adaptations occurring in denervated but otherwise healthy brain regions. It is tempting to speculate that PAR1-inhibition may protect neuronal networks from alterations in plasticity and could even restore the ability of neurons to adjust structure and function in a homeostatic manner under conditions of pathological PAR1-activation. Indeed, our previous work disclosed that PAR1-activation occludes the ability of neurons to express homeostatic synaptic plasticity (Becker et al., 2014). Hence, neural PAR1-inhibitors may exert beneficial effects: (1) by the well documented neuroprotective effects at primary lesion sites, e.g., by preventing neural cell death and apoptosis; (2) by restoring the ability of neurons to express plasticity in adjacent brain regions exhibiting increased PAR1-activity; and (3) by *not affecting* compensatory structural and functional adaptations in remote denervated and non-denervated brain regions, which are not directly affected by the primary lesion site.

## Author Contributions

GS, CG, AS, MH, and SS acquired and analyzed data. NM and AV conceived this study, designed experiments and wrote the article with help of ML and TD. All authors were involved in data interpretation and critically revising the manuscirpt.

## Funding

Supported by German Israeli Foundation (GIF G-1317-418.13/2015 to NM and AV).

## Conflict of Interest Statement

The authors declare that the research was conducted in the absence of any commercial or financial relationships that could be construed as a potential conflict of interest.
